# A Case of Mitral Annular Disjunction in Marfan Syndrome

**DOI:** 10.1016/j.case.2021.04.002

**Published:** 2021-05-04

**Authors:** John Dawdy, Aditya Sood, Tushar Mishra, Carlos Oviedo, Anupama Kottam, Luis Afonso

**Affiliations:** a Division of Cardiology, Department of Internal Medicine, Detroit Medical Center/Wayne State University, Detroit, Michigan; b Department of Internal Medicine, Detroit Medical Center/Wayne State University, Detroit, Michigan

**Keywords:** Mitral annular disjunction, Mitral valve annulus, Marfan syndrome, Ventricular arrhythmia, Echocardiography

## Abstract

• Mitral annular disjunction is an underrecognized finding on echocardiography.
• Mitral valve prolapse is strongly associated with mitral annular disjunction.
• Patients with Marfan syndrome are potentially high risk for mitral annular disjunction.
• Paradoxical annular unsaddling occurs in mitral annular disjunction.
• Mitral valvular apparatus tension during systole contributes to arrhythmic syndrome.

## Graphical abstract

**Figure:**
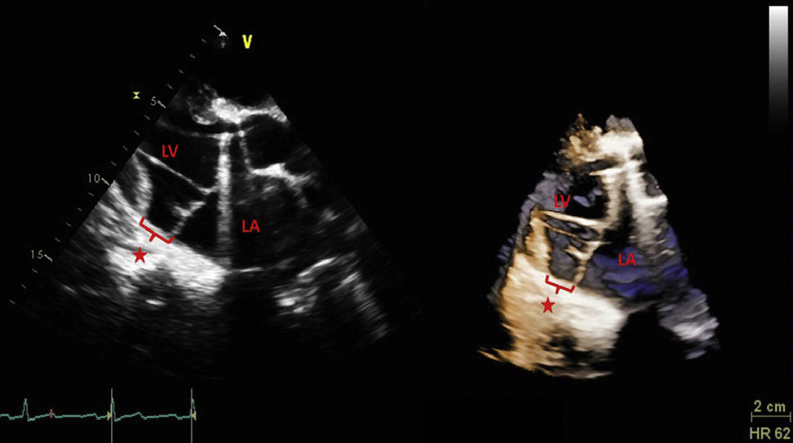

## Introduction

Mitral annular disjunction (MAD) is a clinically relevant finding that is readily detectable but often overlooked on echocardiography. It is an abnormal atrial displacement of the hinge point of the mitral valve away from the ventricular myocardium, identified as separation between the posterior leaflet insertion into the left atrial wall and the base of the left ventricular free wall.^1^ Quantification of MAD has focused predominantly on maximal disjunction distance, but a disjunction index has also been used where the product of the disjunction arc degree and the maximum disjunction distance is calculated in order to give a more robust description of the separation identified.^1^ A disjunction index has been shown to correlate significantly with the effective regurgitant orifice. Most often, MAD is identified using echocardiography in parasternal long-axis and apical four-chamber views in systole, because in diastole the ventricular myocardium appears to be appropriately situated under the annulus. Cardiac magnetic resonance imaging provides another modality for MAD diagnosis, along with providing insight into arrhythmogenic foci of myocardial fibrosis/scarring. Classically, MAD is associated with myxomatous mitral valves and mitral valve prolapse (MVP), although it may be present in their absence. Dejgaard *et al*,^2^ identified MVP in 78% of patients with MAD. Here we present a case of MAD in a subpopulation of patients at increased risk of MVP where MAD has yet to be highlighted.

## Case Presentation

**Figure 1:**
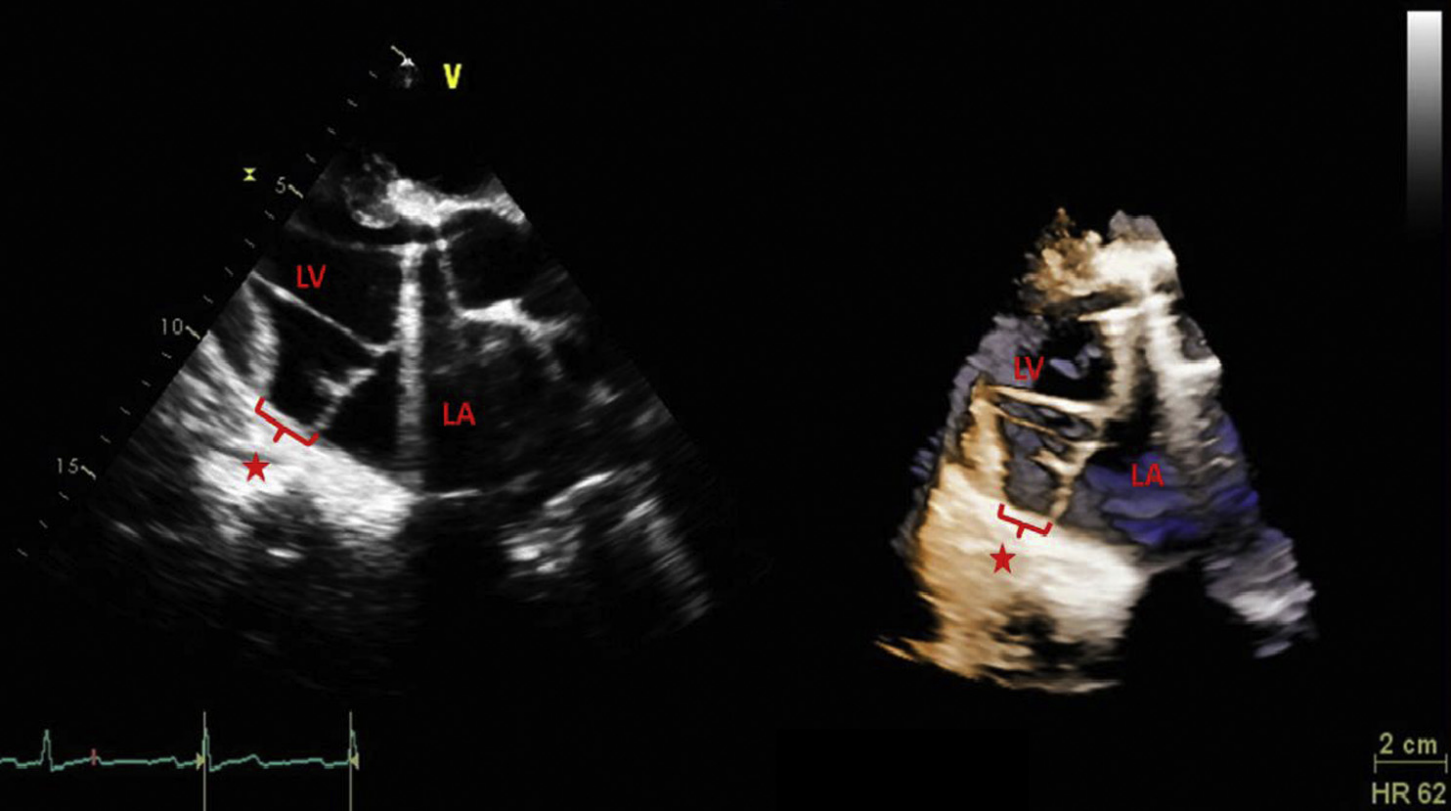
Parasternal long-axis view demonstrating MAD (*red star*) in both two-dimensional **(A)** and three-dimensional **(B)** echocardiography. Maximal disjunction distance is indicated by the red bracket. *LA*, Left atrium; *LV*, left ventricle.

A 23-year-old man with Marfan syndrome along with previous aortic valve replacement and root repair presented after a syncopal episode. He experienced prodromal symptoms including palpitations, tachycardia, and lightheadedness. He had no previous similar episodes or history of arrhythmia. Previous electrocardiogram (ECG) showed sinus rhythm with first degree atrioventricular block and biatrial enlargement. On arrival, he was hypotensive and tachycardic with ECG consistent with atrial fibrillation. Physical exam was remarkable for severe kyphosis and scoliosis, dry oral mucosa, tachycardic irregular rhythm, systolic murmur, tachypnea, and arachnodactyly. Blood pressure and heart rate responded to a 1 L intravenous fluid bolus. Transthoracic echocardiography demonstrated a severe centrally directed mitral regurgitation (MR) with a severely dilated left atrium. Bileaflet MVP with redundant mitral valve leaflets and prominent MAD was apparent with maximal disjunction distance of 19.0 mm (Figure 1, Video 1). Given the patient's severe MR, surgical mitral valve repair was performed, which the patient tolerated without complication. An implantable loop recorder was placed for further arrhythmia monitoring. Six months postoperatively the patient was doing generally well. He had experienced an episode of atrial flutter and had undergone successful electrical cardioversion about 3 months postoperatively. No ventricular arrhythmias have been noted on loop recorder.

## Discussion

The prevalence of MAD in Marfan syndrome has not yet been investigated, but its association with MVP likely goes hand in hand with an association with MAD. The incidence of MVP associated with Marfan syndrome is variable, ranging from 28% to 80%, likely a function of age distribution in the study populations, as notable mitral valve features (MVP, MR) increase with age.

Marfan syndrome is associated with mutations in fibrillin-1 (FN1) on chromosome 15q21.1, although it can also be caused by inactivation mutations in transforming growth factor beta (TGF-β) receptor 2 located at 3p24.2-p25. Ng *et al*^3^ hypothesized that the fibrillin-1-TGF-β pathway is implicated in the pathogenesis of MVP in a murine model of Marfan syndrome and demonstrated a causal relationship between TGF-β dysregulation and abnormal mitral valve structure.

Mitral valve prolapse is generally considered sporadic but is a final common pathway for a variety of genetic and acquired disorders (Marfan syndrome, Ehlers-Danlos syndrome, aneuploidy syndromes, Loeys-Dietz syndrome, pseudoxanthoma elasticum, and Larsen-like syndrome, among others), which presumably weaken the connective tissue of the valve and lead to leaflet elongation, thickening, and degeneration. One case report implicated a truncating variant in the FLNC-encoded filamin C, an actin-binding protein critical for structural integrity of the sarcomere in cardiac and skeletal muscle, as a potential proarrhythmic genetic substrate for arrhythmogenic bileaflet MVP syndrome.^4^

Similar to MVP, MAD has been associated with an arrhythmic syndrome with a high prevalence of ventricular arrhythmia (∼34%) independent of MVP.^2^ Potential markers for ventricular arrhythmia are younger age, previous syncope, more premature ventricular contraction, papillary muscle fibrosis, and larger longitudinal MAD distance in the posterolateral wall assessed by cardiovascular magnetic resonance imaging.^2^ Bains *et al*^4^ proposed a “2-hit” model in which the underlying arrhythmogenic genetic substrate weakens myocyte coupling, which is then exacerbated by excess stress from MVP and/or MAD.

**Figure 2:**
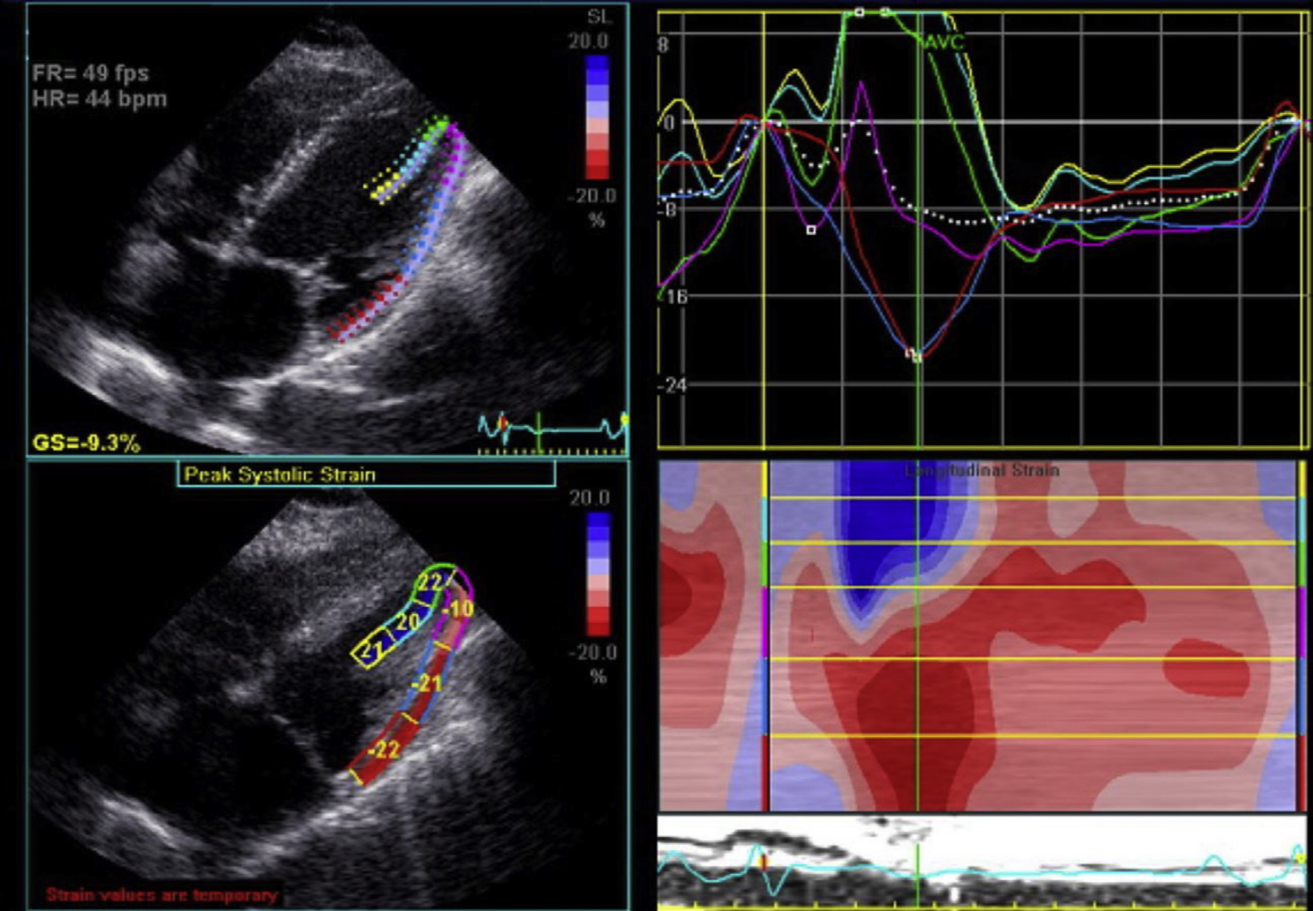
Strain region of interest applied to the papillary muscle to emphasize how valvular unsaddling exaggerates the mechanical stresses on the leaflets and chordae in systole, which may predispose and perpetuate leaflet/chordae degeneration. Note paradoxical systolic lengthening of papillary muscle (*teal and yellow strain curves*).

The normal saddle-shaped structure of the mitral annulus undergoes complex conformational changes through the cardiac cycle in order to provide a balanced distribution of mechanical stresses. In MAD, annular flattening and dilation in systole have been demonstrated, which increase the stress on the leaflets and chordae.^1^ Placement of strain tracing over the papillary muscle demonstrates the paradoxical tension on the valvular apparatus during systole in our patient with MVP and MAD (Figure 2). Measures of papillary muscle strain have been used previously to explore its importance with regard to different etiologies of MR, often using slightly different protocols.^5^^,^^6^ One prior study by Grapsa *et al*^6^ demonstrated that in patients with isolated posterior leaflet MVP, preoperatively the posteromedial papillary muscle experiences increased strain when compared to the anterolateral papillary muscle and that this strain is reduced and near normalized between the papillary muscles after mitral valve repair; the investigators hypothesize that the initial asymmetric tension is exerted by the respective prolapsing leaflet.^6^ In our case, we use a strain tracing that includes the lateral wall of the left ventricle to demonstrate that while shortening of the lateral wall segments occurs, the papillary muscle experiences lengthening with increased tension. These altered conformational changes have been labeled paradoxical annular “unsaddling” and are explained by a functional decoupling of the annular and ventricular motions.^1^ The increased mechanical stress on the leaflets and chordae likely contributes to the scarring/fibrosis seen in the papillary muscles and basal lateral left ventricular myocardium that acts as a nidus for arrhythmia.

No specific diagnostic or treatment guidelines exist for MAD. Ambulatory ECG monitoring and serial echocardiographic monitoring have been suggested without specifics. The role of pharmaceutical and device therapy in these patients remains uncertain at this time but should be a direction of future research. In a case by Bains *et al*,^4^ focal ablation was used to reduce premature ventricular contraction burden in a patient with arrhythmogenic bileaflet MVP syndrome with success.

The currently proposed mechanism of the arrhythmic syndrome suggests that surgical correction could play a role in limiting further arrhythmic risk. Without addressing MAD, annular-ventricular decoupling may persist and impact sustained surgical outcomes. A modified mitral valve repair technique has been used in some institutions in recognition of this.^7^ Similarly, transcatheter mitral valve repair targeting leaflet pathology (e.g., MitraClip) may not fully address the annular dysfunction caused by disjunction.

## Conclusion

The dynamic nature of MAD contributes to its difficult identification, and a concerted effort must be made in order to identify this condition, which is known to increase the risk of ventricular arrhythmia. We highlight a case of MAD in Marfan syndrome in order to emphasize the increased risk in this population along with other syndromes associated with increased risk of MVP and malignant arrhythmias. This subset of patients is already predisposed to aortic dissection, and the presence of MAD may represent yet another albeit lesser recognized risk factor for sudden cardiac death. We also provide a visualization of the mechanical stress applied to the papillary muscles, potentially contributing to the underlying mechanism of the arrhythmic syndrome associated with this condition.

## Supplementary Data

Video 1 Parasternal long-axis view demonstrating bileaflet MVP with redundant mitral valve leaflets and prominent MAD in both two-dimensional and three-dimensional echocardiography.

## References

[1] Lee A.P., Jin C.N., Fan Y., Wong R.H.L., Underwood M.J., Wan S. (2017). Functional implication of mitral annular disjunction in mitral valve prolapse: a quantitative dynamic 3D echocardiographic study. JACC Cardiovasc Imaging.

[2] Dejgaard L.A., Skjolsvik E.T., Lie O.H., Ribe M., Stokke M.K., Hegbom F. (2018). The mitral annulus disjunction arrhythmic syndrome. J Am Coll Cardiol.

[3] Ng C.M., Cheng A., Myers L.A., Martinez-Murillo F., Jie C., Bedja D. (2004). TGF-beta-dependent pathogenesis of mitral valve prolapse in a mouse model of Marfan syndrome. J Clin Invest.

[4] Bains S., Tester D.J., Asirvatham S.J., Noseworthy P.A., Ackerman M.J., Giudicessi J.R. (2019). A novel truncating variant in FLNC-encoded filamin C may serve as a proarrhythmic genetic substrate for arrhythmogenic bileaflet mitral valve prolapse syndrome. Mayo Clin Proc.

[5] Tigen K., Karaahmet T., Dundar C., Guler A., Cevik C., Basaran O. (2010). The importance of papillary muscle dyssynchrony in predicting the severity of functional mitral regurgitation in patients with non-ischaemic dilated cardiomyopathy: a two-dimensional speckle-tracking echocardiography study. Eur J Echocardiogr.

[6] Grapsa J., Zimbarra Cabrita I., Jakaj G., Ntalarizou E., Serapheim A., Demir O.M. (2015). Strain balance of papillary muscles as a prerequisite for successful mitral valve repair in patients with mitral valve prolapse due to fibroelastic deficiency. Eur Heart J Cardiovasc Imaging.

[7] Eriksson M.J., Bitkover C.Y., Omran A.S., David T.E., Ivanov J., Ali M.J. (2005). Mitral annular disjunction in advanced myxomatous mitral valve disease: echocardiographic detection and surgical correction. J Am Soc Echocardiogr.

